# Comparative Analysis of Plastomes of *Artemisia* and Insights into the Infra-Generic Phylogenetic Relationships Within the Genus

**DOI:** 10.3390/genes16060659

**Published:** 2025-05-29

**Authors:** Xinqiang Guo, Weiquan Huang, Zhiyi Zhao, Dawei Xue, Yuhuan Wu

**Affiliations:** 1 College of Life and Environmental Sciences, Hangzhou Normal University, Hangzhou 311121, China; xqguo@hznu.edu.cn (X.G.); huangweiquan@stu.hznu.edu.cn (W.H.); 2022210301055@stu.hznu.edu.cn (Z.Z.); dwxue@hznu.edu.cn (D.X.); 2 Zhejiang Provincial Key Laboratory for Genetic Improvement and Quality Control of Medicinal Plants, Hangzhou Normal University, Hangzhou 311121, China

**Keywords:** *Artemisia*, Asteraceae, chloroplast, phylogenomics, plastome

## Abstract

**Background**: *Artemisia* is a large and complex genus comprising about 500 species. Currently, only a limited number of plastomes (the chloroplast genome) of *Artemisia* are available. Their structures have not been comparatively analyzed, and a phylogenetic backbone based on plastome-scale data is still lacking. This situation has greatly hindered our understanding of the plastome variation patterns and infra-generic relationships of the genus. **Methods**: We newly sequenced 34 *Artemisia* plastomes representing 30 species and three varieties. Combining this with previously published plastomes, we comparatively analyzed their structure and constructed phylogenetic relationships using the protein-coding sequences (CDS) of plastomes. **Results**: Our analyses indicated that the *Artemisia* plastomes are conserved in terms of their structure, GC content, gene number, and order. The sequence divergence is higher in the LSC and SSC regions than in the IR regions. Three protein-coding genes and four non-coding regions, i.e., *accD*, *petG*, *ycf1*, *rpoC1-rpoC2*, *rpoC2-rps2*, *trnG(UCC)-trnfM(CAU)*, and *ndhG-ndhI*, were highly diverse and could be chosen as candidates of DNA barcodes. Phylogenetic trees were divided into several clades, and all four main subgenera were not monophyletic. Additionally, the phylogenetic position of *A. stracheyi* is still controversial. **Conclusions**: Plastomes can provide important information for phylogenetic constructions. This study provides insights into the infra-generic relationships within *Artemisia* and also lays a foundation for future evolutionary studies of this genus.

## 1. Introduction

*Artemisia* is the largest genus in the tribe Anthemideae of Asteraceae, comprising about 500 species [1,2,3,4]. Members of this genus are mainly distributed in temperate regions of the Northern Hemisphere, with a few species occurring in Africa, South America, and the Hawaiian Islands [1,2,3,4,5,6]. Members of this genus, i.e., *A. argyi* H. Lév. & Vaniot and *A. capillaris* Thunb. have been widely used as traditional herbal remedies in China, and some species have broad applications as food or forage. The most famous one is undoubtedly *A*. *annua* L. Youyou Tu discovered the anti-malaria artemisinin from this species and was awarded the Nobel Prize in Physiology or Medicine in 2015 [7,8,9]. This genus thus receives extensive scientific attention, especially in the fields of phytochemistry and pharmacology.

*Artemisia* represents one of the most notoriously difficult groups in plant taxonomy, largely due to the complex variation patterns of characters [2,3]. Historically, morphological characters were widely used to divide taxa and unravel the relationships within the genus. This has resulted in continuous taxonomic rearrangements [1,2,3,4,10,11,12,13,14,15,16]. Infra-generic classifications divided the genus into subgenera, but sections or series were also consecutively proposed. Among them, the generally accepted one comprises four subgenera, including subg. *Absinthium* (Miller) Less., subg. *Artemisia*, subg. *Dracunculus* (Besser) Rydb., and subg. *Seriphidium* Besser ex Less., and are mainly based on the type of capitula, fertility of disk florets, and hairy receptacles [3,4]. However, phylogenetic studies using a limited number of molecular markers (e.g., ITS, *psbA*–*trnH*, *rpl32–trnH*, *trnL–trnF*, and *trnS–trnC*) revealed that the infra-generic phylogenetic relationships of *Artemisia* were, to some degree, incongruent with the divisions based on morphological characters [5,17,18,19,20]. Subg. *Absinthium*, subg. *Dracunculus*, and subg. *Seriphidium* were not monophyletic. Species previously assigned to subg. *Artemisia* were even scattered into several clades. Furthermore, subg. *Tridentatae* (Rydb.) McArthur and subg. *Pacifica* Hobbs & Baldwin were proposed so as to accommodate some species occurring in America and three Hawaiian endemic species (*A*. *australis*, *A. kauaiensis*, and *A. mauiensis*) together with *A. chinensis*, respectively [5]. Using nuclear single-nucleotide polymorphism (SNP) data that were obtained by genome-skimming sequencing technology, Jiao et al. reconstructed a phylogeny for *Artemisia* consisting of eight main clades. Accordingly, they proposed a revised clade-based infra-generic classification, dividing the genus into eight subgenera [21]. To some extent, the discordance about the infra-generic relationships reflects the complex evolutionary history of *Artemisia*.

Plastids (chloroplasts), commonly found in plants, are important in plant growth and development [22,23,24]. Typically, the plastome (chloroplast genome) is a closed loop with a quadripartite structure comprising a large single-copy region (LSC), a small single-copy region (SSC), and two inverted repeat regions (IRa and IRb). In angiosperms, the length usually ranges from 120–200 kb. Each genome tends to contain approximately 80 protein-coding genes, four rRNAs, and 30 tRNAs [25,26]. Due to its small size, uniparental inheritance, conserved sequence and structure, and high cellular copy numbers, the plastome has been an advantageous resource for various evolutionary studies [27]. Some plastid genes (*rbcL*, *matK*) have been extensively used to estimate phylogenetic relationships at deep and shallow levels [26,27,28,29,30]. Some faster-evolving genes (i.e., *matK*, *ndhF*, *rbcL*, and *rpoC1*) and spacer regions (i.e., *atpF-atpH*, *psbK-psbI*, and *trnH-psbA*) have been developed as DNA barcode markers to identify taxa [31]. In *Artemisia*, a few plastid regions, including *psbA–trnH*, *rpl32–trnH*, and *trnS–trnC*, usually concatenated with nuclear regions (ITS and ETS), were used to construct generic or infra-generic phylogenetic relationships and explore the evolutionary history of the genus [5,6,7,17,18,19,20,32].

With the advancement of next-generation sequencing (NGS) technology and the decrease in sequencing costs, it is becoming easier to obtain complete plastome sequences. The plastome data have exhibited greater potential for resolving challenging phylogenetic relationships in a wide spectrum of plant lineages, e.g., *Eriocaulon* L. (Eriocaulaceae), *Trigonotis* Steven (Boraginaceae), Apocynaceae, and Ophioglossaceae [26,27,28,29,30,33,34]. Numerous historically difficult issues in plant phylogenetics have been satisfactorily addressed, indicating the indispensable role played by plastomes. For *Artemisia*, Kim et al. first conducted a comparative analysis of the plastomes of 32 *Artemisia* species in East Asia [35]. The study revealed that the coding sequences of *accD* and *ycf1* were under weak positive selection and highly variable. The plastomes were sufficiently polymorphic for use as super-barcodes [35]. They further confirmed that subg. *Artemisia* was not monophyletic. Using a plastome data matrix of 38 species, including 18 species from subg. *Seriphidium*, Jin et al. found that subg. *Seriphidium* was inserted into the main clade of *Artemisia* and segregated into two main clades [36]. Furthermore, their structural analysis indicated that the plastomes are relatively conserved, with some variations only in the IR borders. At the National Center for Biotechnology Information (NCBI), only a limited number of plastomes of *Artemisia* are available. In contrast with the large number of taxa in the genus, the percentage of sequenced plastomes did not match the *Artemisia* biodiversity well. There are still gaps in our knowledge of the general variation pattern of *Artemisia* plastomes, especially regarding their structure, gene order, IR/SC boundary, and IR expansion. A finer-scale phylogenetic relationship, constructed using plastid data with more informative characters and denser taxon sampling, is still lacking. Comparisons between phylogenies constructed using data from nuclear DNA and plastome to explore cyto-nuclear (i.e., chloroplast–nuclear) discordance are also needed.

Considering this situation, we newly sequenced, assembled, and annotated 34 *Artemisia* plastomes representing 30 species and three varieties in this study. Combining this with previously published *Artemisia* plastomes from a public database, we conducted comparative analyses and constructed phylogenies in order to (1) study the plastome variation patterns of this genus, (2) identify variable regions as DNA barcode candidates for future taxa identification, and (3) recover the backbone of the *Artemisia* phylogeny using a plastome-scale dataset. Overall, this study will improve our knowledge of *Artemisia* plastomes, provide potential genetic markers for taxa identification, and also advance our understanding of the phylogenetic relationships within the genus.

## 2. Materials and Methods

### 2.1. Taxa Sampling, DNA Extraction, and Sequencing

In this study, we newly sequenced 34 plastomes representing 30 species and three varieties of *Artemisia* (Table 1). Detailed information on the taxon, voucher specimen, and collection locality is provided in Table S1. All the materials were collected during our field trips in China. The voucher specimens were all identified by Xinqiang Guo, the first author of this study, and deposited in the Herbarium of South China Botanical Garden, Chinese Academy of Sciences (IBSC). Additionally, we also downloaded 38 *Artemisia* plastomes (representing 34 species and one form) from the NCBI Genbank database (https://www.ncbi.nlm.nih.gov/nuccore/, accessed on 1 May 2024) (Table S2). A total of 72 plastomes of *Artemisia* were obtained and used in a comparative plastome analysis. For phylogenetic reconstruction, three plastomes representing three species of *Ajania* (*A. fruticulosa* (Ledeb.) Poljak., *A. nematoloba* (Hand.-Mazz.) Ling & Shih, *A. khartensis* (Dunn.) Shih), and *Chrysanthemum przewalskii* (Poljakov) H. Ohashi & Yonek., were selected as outgroups.

Fresh leaves were dried with silica gel and kept in a refrigerator. High-quality total genomic DNA of the plant samples was extracted from 10 mg of silica gel-dried leaves using a modified CTAB (cetyl trimethyl ammonium bromide) DNA extraction method [37]. The DNA samples were sent to Shanghai Personal Biotechnology Co., Ltd. (Shanghai, China), and a 150 bp paired-ended library with an average insert size of approximately 400 bp was prepared according to the manufacturer’s manual (Illumina, San Diego, CA, USA). Shotgun sequencing was performed on the Illumina NovaSeq 6000 platform. Approximately 3 Gb of raw reads were generated for each sample.

**Table 1:** Summary of the 34 plastomes newly sequenced in this study.

Taxon	Genbank Accession Number	Nucleotide Length (bp)				Number of Genes			GC Content
		Total	LSC	SSC	IR	Protein-Coding Genes	rRNA Genes	tRNA Genes	(%)
*A. sichuanensis*	PP898074	151,230	82,935	18,375	24,960	87	37	8	37.50%
*A. adamsii*	PP898105	151,231	82,955	18,337	24,969	87	37	8	37.40%
*A. bhutanica*	PP898085	151,269	82,949	18,398	24,961	87	37	8	37.50%
*A. blepharolepis*	PP898101	150,908	82,963	18,019	24,963	87	37	8	37.50%
*A. comaiensis*	PP898079	151,298	82,930	18,448	24,960	87	37	8	37.50%
*A. fulgens* var. *meiguensis*	PP898075	151,201	82,856	18,425	24,960	87	37	8	37.50%
*A. gyitangensis*	PP898091	151,199	82,810	18,471	24,959	87	37	8	37.50%
*A. integrifolia*	PP898104	151,117	82,912	18,285	24,960	87	37	8	37.40%
*A. jilongensis*	PP898086	151,275	82,538	18,883	24,927	87	37	8	37.50%
*A. linyoureunensis*	PP898096	151,174	82,718	18,594	24,931	87	37	8	37.50%
*A. mairei*	PP898080	151,045	82,911	18,200	24,967	87	37	8	37.50%
*A. mattfeldii* var. *etomentosa*	PP898076	151,087	82,929	18,238	24,960	87	37	8	37.50%
*A. minor*	PP898083	151,083	82,556	18,673	24,927	87	37	8	37.50%
*A. mongolica*	PP898103	151,193	82,795	18,478	24,960	87	37	8	37.50%
*A. neosinensis*	PP898094	151,172	82,832	18,422	24,959	87	37	8	37.50%
*A. nortonii*	PP898084	151,028	82,873	18,235	24,960	87	37	8	37.50%
*A. phyllobotrys*	PP898077	151,171	82,931	18,312	24,964	87	37	8	37.50%
*A. qinlingensis*	PP898098	151,181	82,921	18,326	24,967	87	37	8	37.50%
*A. selengensis*-1	PP898102	151,228	82,902	18,406	24,960	87	37	8	37.50%
*A. sericea*	PP898073	150,870	82,852	18,094	24,962	87	37	8	37.50%
*A. smithii*	PP898097	151,225	83,037	18,272	24,958	87	37	8	37.40%
*A. stracheyi*	PP898081	151,327	82,937	18,470	24,960	87	37	8	37.50%
*A. stricta*	PP898082	150,770	82,911	17,939	24,960	87	37	8	37.50%
*A. sylvatica*	PP898100	151,200	82,982	18,296	24,961	87	37	8	37.40%
*A. tafellii*	PP898088	151,274	82,937	18,417	24,960	87	37	8	37.50%
*A. tangutica*	PP898099	151,191	82,833	18,452	24,953	87	37	8	37.50%
*A. tournefortiana*	PP898072	151,106	82,600	18,606	24,950	87	37	8	37.50%
*A. tridactyla*	PP898095	150,654	82,810	17,946	24,949	87	37	8	37.50%
*A. viscidissima*	PP898078	151,241	82,655	18,622	24,982	87	37	8	37.50%
*A. waltonii*	PP898087	151,038	82,909	18,209	24,960	87	37	8	37.50%
*A. waltonii* var. *yushuensis*	PP898092	150,636	82,981	17,735	24,960	87	37	8	37.40%
*A. youngii*	PP898089	151,249	82,948	18,363	24,969	87	37	8	37.40%
*A. yunnanensi*-1	PP898090	151,190	82,987	18,283	24,960	87	37	8	37.40%
*A. yunnanensis*-2	PP898093	151,232	82,977	18,335	24,960	87	37	8	37.40%

### 2.2. Plastome Assembly and Annotation

Trimmomatic v.0.40 [38] was used to remove adapters and filter low-quality reads. NOVOPlasty v2.5.9 [39] and GetOrganelle pipeline v1.7.2 [40] were used to de novo assemble plastomes with the suggested default parameters, with the complete plastome sequence of *A. annua* (Genbank Accession Number: KY085890.1) used as a reference. The obtained scaffolds were checked using Bandage v0.8.1 [41]. Assembled plastomes were annotated by using the program GeSeq [42] and Plastid Genome Annotator (PGA) [43], with the annotated plastome of *A*. *annua* (GenBank Accession Number: KY085890.1) used as a reference. To precisely define the start and stop codons, intron boundaries, and tRNA genes, annotations were manually adjusted according to the reference plastome in Geneious Primer v2021.0.3 (Biomatters Ltd., Auckland, New Zealand). The 34 newly sequenced plastomes were deposited into the GenBank database (Table S1). The raw data and plastome sequences downloaded from NCBI were reassembled and reannotated following the procedures of the newly sequenced samples. The circular plastid genome maps were visualized using OrganellarGenome DRAW v1.3.1 [44].

### 2.3. Comparative Analyses, Identification of Divergence Hotspots, and Simple Sequence Repeats

We selected 23 plastomes (representing 22 species and one variety) of *Artemisia* to conduct plastome comparisons using the mVISTA program with the Shuffle-LAGAN model [45]. The annotation of *A. sieversiana* (GenBank Accession Number: ON729303) was chosen as a reference. To identify potential hotspots of nucleotide diversity in 72 *Artemisia* plastomes, the protein-coding sequences (CDSs) and intergeneric regions were extracted, respectively, and aligned using MUSCLE v.3.8.31 [46] with the default parameters. Then, the nucleotide diversity was estimated using DnaSP v.6 [47], with the window length set as the whole length of each matrix (Table S3). To have a comprehensive overview of the IR expansion or contraction in the plastomes of *Artemisia*, we selected 66 plastomes and visualized the borders of the IR/SC regions using CPJSdraw v1.0.0 [48]. Simple sequence repeats (SSRs) of 72 plastomes (Table S4) were identified using MISA-web (MicroSAtellite; https://pgrc.ipk-gatersleben.de/misa/, accessed on 1 May 2024) [49] with the threshold repeat numbers of 10, 5, 4, 3, 3, and 3 for the mono-, di-, tri-, tetra-, penta-, and hexa-nucleotides, respectively.

### 2.4. Phylogenetic Analysis

The CDSs of 76 plastomes, including 72 of *Artemisia*, three of *Ajania* (*A*. *fruticulosa*, *A*. *nematoloba*, *A*. *khartensis*), and one of *Chrysanthemum* (*C. przewalskii*), were extracted using PhyloSuit v7.3.1 [50] and Geneious Primer v2021.0.3. The datasets were aligned using MUSCLE v. 3.8.31 [46] and manually adjusted using AliView v1.26 [51]. All the individual CDS matrices were concatenated into a single supermatrix using Geneious Primer v2021.0.3.

PartitionFinder 2 [52] was used to determine the best-fit partitioning scheme and the most suitable substitution model. Bayesian phylogenies were constructed using MrBayes v3.2.7a [53]. Two parallel analyses, each with four chains (one cold and three hot chains), were run for 40 million Markov Chain Monte Carlo (MCMC) generations, with the trees being sampled every 1000 generations. The first 25% of the sampled trees were discarded as burn-ins. The remaining trees were used to estimate the posterior probabilities (PP). Tracer v.1.6 [54] was used to ensure convergence and adequate sampling with the average standard deviation of split frequencies < 0.01 and effective sample sizes (ESS) of all parameters > 200. The maximum likelihood (ML) analysis was carried out in RAxML-HPC v8.2.12 [55] with 1000 bootstrap replicates using a fast bootstrapping algorithm (MLBS) to assess node support. Bootstrap percentage (MLBS and MPBS) values of ≥70 and PP values of ≥0.95 were regarded as strong support. The final tree files were visualized in FigTree v1.4.3 (https://tree.bio.ed.ac.uk/software/figtree/, accessed on 1 May 2024) and TreeGraph v2.15.0-887 beta [56].

## 3. Results

### 3.1. Plastome Features of Artemisia Species

**Figure 1:**
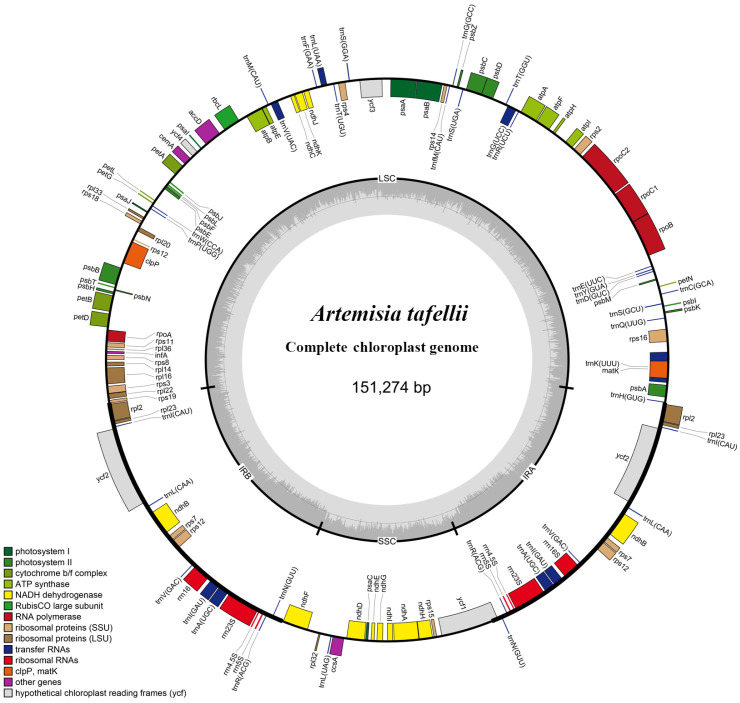
Gene map of the plastome of *Artemisia tafellii*. Genes shown inside the circle are transcribed clockwise, and those shown outside are transcribed counterclockwise. Genes belonging to different functional groups are shown in different colors. The darker gray color in the inner circle corresponds to the GC content, and the lighter gray color corresponds to the AT content. IR, inverted repeat region; LSC, large single copy; SSC, small single copy.

A total of 72 *Artemisia* plastomes were included in this study, representing 63 species, three varieties, and one form (Table 1, Tables S1 and S2). Among them, 34 plastomes of 30 species and three varieties were newly sequenced and assembled in this study (Table 1 and Table S1). All plastomes showed a typical quadripartite structure, comprising a large single-copy (LSC) region, a small single-copy (SSC) region, and two inverted repeated (IRa/b) regions (Figure 1). Their sizes ranged from 150,586 bp (*A. ferganensis*) to 151,327 bp (*A. stracheyi*), with a difference of 741 bp and a mean length of 151,108 bp. The lengths of the LSC, SSC, and IR regions were 82,313–83,061 bp, 17,735–18,883 bp, and 24,927–24,985 bp, respectively. The total GC content ranged from 37.40% to 37.51%, with a mean value of 37.50%. The gene categories were rather conserved. A total of 132 genes, including 87 protein-coding genes, 37 tRNA genes, and eight rRNA genes, were included in every plastome. Detailed information on these plastomes is provided in Table 1, Tables S1 and S2.

**Figure 2:**
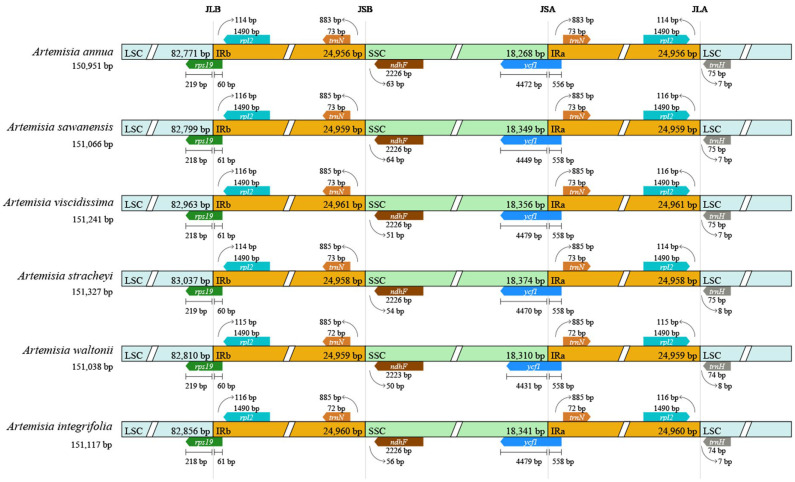
Comparison of the borders of large single-copy (LSC), inverted repeat (IR), and small single-copy (SSC) regions among six *Artemisia* plastomes. JLB (IRb /LSC), JSB (IRb/SSC), JSA (SSC/IRa), and JLA (IRa/LSC) denote the JSs between each corresponding region in the plastome.

The boundaries between the IR and SC regions were compared in 66 *Artemisia* plastomes that represented 62 species, three varieties, and one form. All of them have the same type of SC/IR junctions (Figure 2 and Figure S1). The LSC/IRb junction borders (JLB) were located in the gene *rps19*, with four types recovered. The length of *rps19* in the LSC was 207–219 bp and 60–72 bp in the IRb region. The dominant type was 219 bp in the LSC region and 60 bp in the IRb region. The SSC/IRa junction borders (JSA) were located in the gene *ycf1*, with 4428–4500 bp in the SSC region and 556–565 bp in the IRa region. At the IRb/SSC junction borders (JSB), the distances between the gene *ndhF* and the border range were 42–82 bp. At the LSC/IRa junction borders (JLA), the distances between the gene *trnH* and the border range were 2–135 bp. The IR regions were highly conserved and similar in length and structure. Additionally, no gene rearrangements, inversions, or losses among these plastomes were found.

### 3.2. Plastome Sequence Divergence

**Figure 3:**
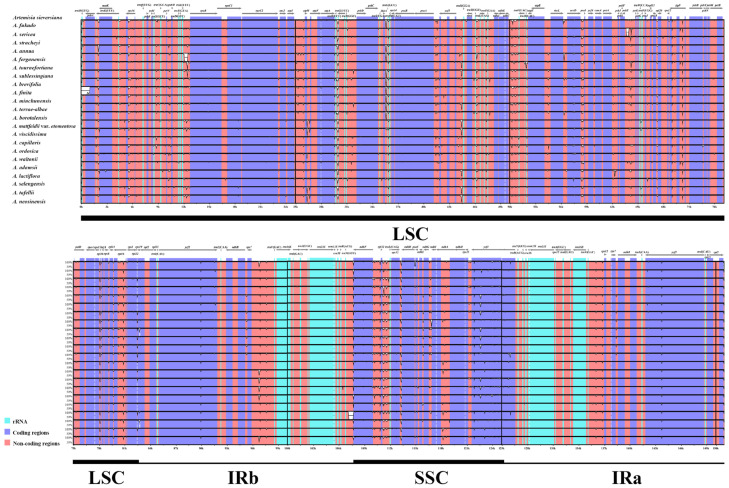
Sequence alignment of 23 *Artemisia* plastomes using the mVISTA program with *A. sieversiana* used as a reference. The X-axis indicates the sequence length, and the Y-axis indicates the percentage identity, ranging from 50 to 100%. The gray arrows below the genes denote the gene orientation. The bars below the X-axis show the gene position of the plastome region.

**Figure 4:**
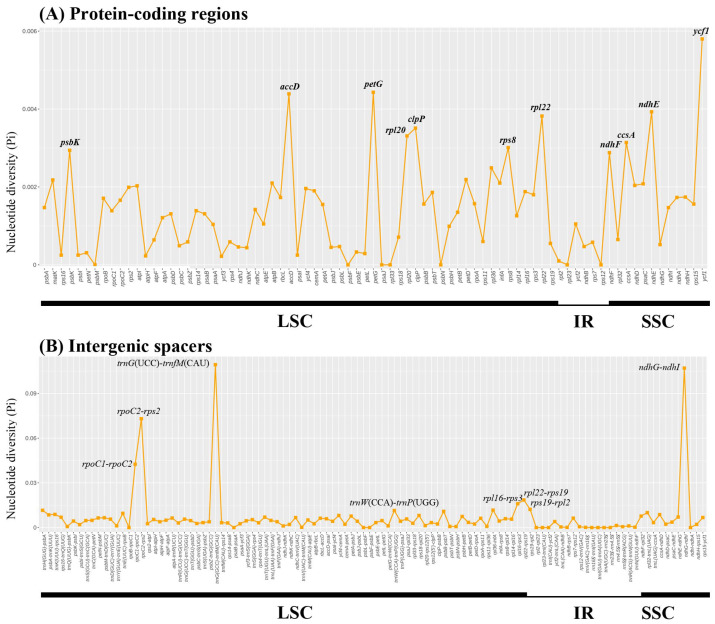
The nucleotide variability (Pi) values in the 72 *Artemisia* plastomes: (**A**) intergenic regions; (**B**) protein-coding genes. These regions are arranged according to their location in the plastome.

The sequence divergence of 23 plastomes was analyzed using the mVISTA program, with *A. sieversiana* (Genbank accession number: ON729303) used as a reference. The plastomes of *Artemisia* were conserved (Figure 3), and the generic regions were more conserved than the intergenic spacer regions. In the LSC and SSC regions, sequence divergence was higher than that in the IR regions. The nucleotide polymorphism (Pi) values showed very similar results on sequence divergence (Figure 4). Most of the CDSs were conserved, with Pi values lower than 0.002. Only three genes (*accD*, *petG*, and *ycf1*) had Pi values between 0.004 and 0.006, and nine CDSs had Pi values between 0.002 and 0.004. Most of the genes with high Pi values (≥0.002) were located in the single-copy regions (Figure 4A, Table S3). The non-coding regions exhibited higher nucleotide variability (Figure 4B, Table S3). The regions *ndhG*-*ndhI*, *trnG*(UCC)-*trnfM*(CAU), and *rpoC2*-*rps2* had Pi values higher than 0.06; the Pi value of *rpoC1*-*rpoC2* was between 0.03 and 0.04, and the others had Pi values lower than 0.03. In the IR regions, the non-coding regions were highly conserved.

### 3.3. Simple Sequence Repeats (SSRs) in Artemisia Plastomes

**Figure 5:**
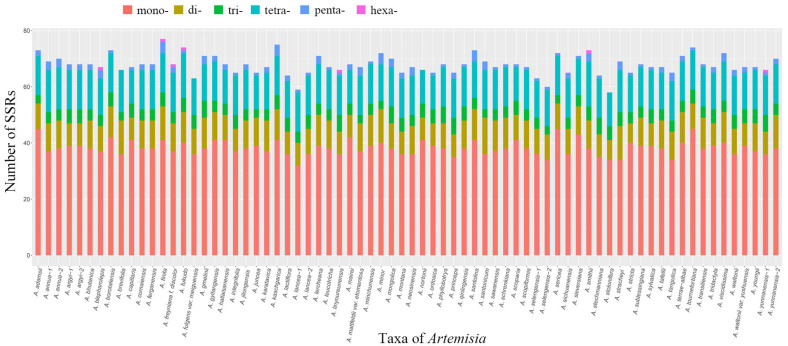
The number of simple sequence repeats (SSRs) in the plastomes of *Artemisia* species. Note: mono-, mono-nucleotides; di-, di-nucleotides; tri-, tri-nucleotides; tetra-, tetra-nucleotides; penta-, penta-nucleotides; hexa-, hexa-nucleotides.

Repeated DNA sequences are important in genome rearrangement. We investigated simple sequence repeats (SSRs) in the alignment of 72 plastomes of *Artemisia*. A total of 4886 SSRs were detected. The number of SSRs varied from 58 to 77 in each plastome. Four plastomes (i.e., *A*. *finite* Kitag., *A. kaschgarica* Krasch., *A. fukudo* Makino, and *A. tournefortiana* Rchb.) had more SSRs than the others (Figure 5, Table S4). Mono-nucleotide repeats are the most abundant (2754, 56.4%), followed by the tetra- (987, 20.2%), di- (692, 14.2%), tri- (318, 6.5%), penta- (128, 2.6%), and hexa-nucleotide (7, 0.1%) repeats (Table S4). The mono-, di-, tri-, and tetra-nucleotide repeats were found in all plastomes, while penta-nucleotide repeats were found in 67 plastomes. In only seven plastomes (representing six species and one form), including *A. blepharolepis* Bunge, *A. finita*, *A. freyniana* f. *discolor* (Kom.) Kitag., *A. fukudo*, *A. linyoureunensis* L. Shultz & Boufford, *A. smithii* Mattf., and *A. yunnanensis* Jeffrey ex Diels, hexa-nucleotide repeats were found. Most SSRs were located in single-copy regions, with 3794 found in the LSC and 673 in the SSC regions. Only 418 SSRs were in the IR regions. Mono-nucleotide repeats may play an important role in genetic variation than other SSR types. The A/T repeats account for nearly 97.8% of the mono-nucleotide repeats, and this result is similar to other studies. Di-nucleotide repeats are represented only by the AT/TA motif. Detailed information on the SSRs in each plastome is provided in Supplementary Table S4.

### 3.4. Phylogenetic Analysis

**Figure 6:**
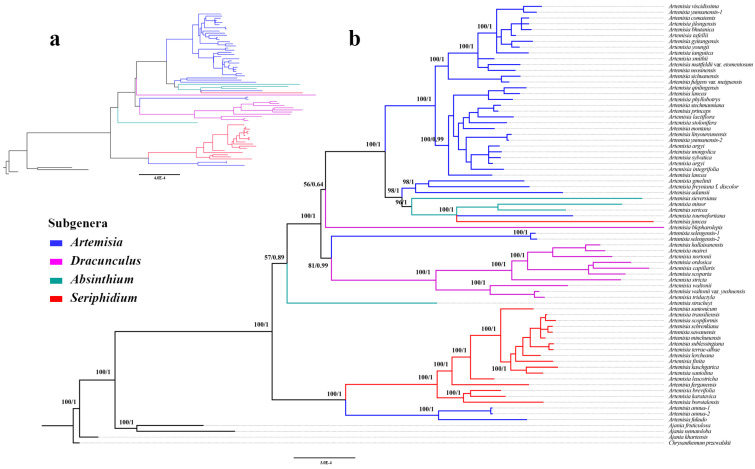
Phylogeny of *Artemisia* based on 79 protein-coding sequences (CDS) of 72 *Artemisia* species and four outgroups: (**a**) Consensus phylogenetic tree reconstructed by Bayesian inference (BI) analysis. The numbers near the branches are the bootstrap support (BS) percentages, obtained from maximum likelihood inference, and the posterior probabilities (PP), obtained from Bayesian analysis (BS/PP). (**b**) A phylogenetic backbone reconstructed by maximum likelihood (ML) inference.

The topologies of the phylogenetic trees constructed from the maximum likelihood (ML) and Bayesian inference (BI) methods were basically similar (Figure 6, Figures S2 and S3). All samples of *Artemisia* were clustered into one single clade, which was a sister to the outgroup *Ajania*–*Chrysanthemum* clade. The genus *Artemisia* was split into two clusters. The basal one (here referred to as Clade 1) further divides into two well-supported (ML bootstrap value (BS) = 100%; Bayesian posterior probabilities (PP) = 1) subclades, with one subclade comprising two samples of *A. annua* and one of *A. fukudo*, and another subclade comprising 17 of subg. *Seriphidium*. Another cluster (here referred to as Clade 2) divides into three main subclades, including one subclade comprising only *A. stracheyi*, one comprising ten samples of subg. *Dracunculus*, and two of *A. selengensis* of subg. *Artemisia*, while the remaining subclade comprises all other samples. However, Clade 2 was not strongly supported (BS = 81, PP = 0.89).

Consistent with previous phylogenetic studies using nuclear markers, our results also confirmed that all four subgenera sampled in this study, i.e., subg. *Absinthium*, subg. *Artemisia*, subg. *Dracunculus*, and subg. *Seriphidium* were not monophyletic (Figure 6, Figures S2 and S3). Most of the species of subg. *Artemisia* were clustered into a monophyletic group, and the other species were inserted into several clades. The phylogenetic position of *A. juncea* of subg. *Seriphidium* was not well resolved. The remaining species of subg. *Seriphidium* formed a monophyletic group. And the subg. *Dracunculus* was also monophyletic when *A. blepharolepis* was excluded. Only three species of subg. *Absinthium* was sampled, including *A. sieversiana* Ehrhart ex Willd., *A. minor* Jacquem. ex Besser, and *A. sericea* Weber ex Stechm., and they formed one clade with *A. juncea* of subg. *Seriphidium* and *A. tournefortiana* of subg. *Artemisia*.

## 4. Discussion

### 4.1. Characteristics of Plastomes and Genetic Variations in Artemisia

To have a better understanding of the variation patterns of the structure of *Artemisia* plastomes, a denser sampling within the genus is inevitable. In this study, a total of 72 plastomes were comparatively analyzed. Consistent with previous studies, the plastomes of *Artemisia* showed a high degree of similarity in terms of the GC content, configuration, gene number, and order [35,36,57,58]. The GC content variation in the genomes is a key feature of genomic organization and strongly varies between species. It is usually associated with the fundamental elements of genome organization, e.g., recombination [59,60,61,62,63]. In the plastomes of *Artemisia*, the GC content did not vary significantly between different species, ranging from 37.40% to 37.51%, which is typical in the plastomes of angiosperm [61]. No rearrangement has been found in these samples. This also reflects that *Artemisia* plastomes are very conservative. In general, the length of *Artemisia* plastomes also falls within the average length range of eudicots [22]. Sequence length uniformity was found between different samples of the same species. For example, two plastomes of *A. annua* were both 150952 bp. It is more common that different samples of the same species have different sequence lengths, e.g., *A. argyi*, *A. lancea* Van., and *A. selengensis* Turcz. ex Bess. Three factors have been proposed to drive the difference in plastome length, including intergenic region variations, differences in genes, and the expansion and contraction of IR regions [60]. All the plastomes are quadripartite, containing the same number of genes, including 87 protein-coding, 37 tRNA, and eight rRNA genes. The 66 plastomes analyzed using CPJSdraw belong to the same IR/SC boundary type. The IR regions only varied by 58 bp, while the LSC regions varied by 748 bp, and the SSC regions varied by 148 bp. Thus, the variations in the length of *Artemisia* plastomes were mainly in the LSC regions.

Previous analyses of whole plastomes revealed that the plastid regions *accD*, *ndhF*, *trnT*, *ycf1*, *rpl32-trnL*, *trnE-ropB*, *trnH-psbA*, *trnK-rps16*, *ndhC-trnV*, and *ndhG-ndhI* are highly variable [35,36,57,58]. As pointed out by Shaw et al., the plastid region might not be consistently variable across different groups [64]. In this study, *accD*, *petG*, *ycf1*, *ndhG-ndhI*, *trnG*(UCC)-*trnfM*(CAU), and *rpoC2*-*rps2* have higher variability and were identified as mutational hotspots for *Artemisia* plastomes. Several plastid regions, including *rpl32–trnH* and *trnS–trnC*, have been used to construct the phylogeny of *Artemisia* [19,36]. However, we found that these regions were not the most informative. These regions may have limited power for resolving phylogenetic relationships within the genus. The combination of *rbcL* and *matK* was recommended as a core plant barcode by the CBOL Plant Working Group [31]. But, the Pi values of *rbcL* and *matK* here were both lower than 0.002, indicating they have a rather limited discriminative power in *Artemisia*. Plastid *accD* and *ycf1* are important for plant fitness and leaf development. As observed in other plant groups, the *accD* and *ycf1* genes have high variable nucleotide sequences in the plastomes analyzed in this study. The genus *Artemisia* is morphologically complex, and species identification is rather difficult. These hotspot regions could be developed as DNA barcodes and used to distinguish taxa.

### 4.2. Phylogenetic Relationships of Artemisia

A well-resolved phylogenetic relationship is critical for a better understanding of the evolutionary process of plants at different ranks, especially for the genus *Artemisia*, which is large and morphologically variable [17]. Using protein-coding sequences (CDS) of plastomes, we reconstructed the phylogenetic relationships for *Artemisia* with a broad taxonomic sampling. To some extent, our results are consistent with previous studies and further confirm that the taxonomic divisions based on morphological characters were in conflict with the molecular phylogenetic relationships. All four subgenera sampled here, including subg. *Absinthium*, subg. *Artemisia*, subg. *Dracunculus*, and subg. *Seriphidium*, were not supported as monophyletic. The subg. *Artemisia,* which is the largest genus in *Artemisia*, however, is polyphyletic, with the sampled taxa inserted into several clades. In his treatment of Chinese *Artemisia*, Ling divided subg. *Artemisia* into two sections: sect. *Artemisia* and sect. *Abrotanum*, which is mainly based on the shape of the leaflets [17]. This division was also not supported by our study. The subg. *Seriphidium* was once considered an independent genus, *Seriphidium* (Bess.) Poljak., by only having bisexual florets [2,65]. However, this was also not supported by our study [36]. Species of subg. *Seriphidium* were clustered into two clades. *Artemisia juncea* formed an independent clade, which is a sister to a large clade, including species of subg. *Absinthium* and subg. *Artemisia*. The other species of subg. *Seriphidium* formed a clade (Figure 6). The subg. *Dracunculus* was monophyletic when two samples of *A. selengensis* of subg. *Artemisia* were included.

Additionally, our analyses also revealed that there exists cyto-nuclear phylogenetic discordance, especially in the position of subg. *Dracunculus* and subg. *Seriphidium*. Previous studies using nuclear regions or nuclear single-nucleotide polymorphisms (SNPs) revealed similar phylogenetic topologies of *Artemisia* [21]. The species of subg. *Dracunculus,* together with some species of subg. *Artemisia* constituted the early divergent clade within *Artemisia*. The remaining taxa were further clustered into two main clades. Most species of subg. *Seriphidium,* together with some species of subg. *Absinthium* and subg. *Artemisia* formed a clade sister to another clade that was formed mainly by species of subg. *Artemisia* and subg. *Absinthium.* The phylogenetic relationships constructed using the CDSs of plastomes revealed a somewhat different topology. The earliest diverging clade of *Artemisia* was constituted by all the species of subg. *Seriphidium* except *A. juncea*, together with *A. annua* and *A. fukudo* of subg. *Artemisia*. The other species were clustered into one clade, which could be further divided into two main clades. One includes all samples of subg. *Dracunculus*, excluding *A. blepharolepis*, and two samples of *A. selengensis*, and the other was constituted by most species of subg. *Artemisia* and some species of subg. *Absinthium*, *A. juncea* of subg. *Seriphidium*, and *A. blepharolepis* of subg. *Dracunculus* (Figure 6). Hybridization is a significant biological process in the evolution of plants, often resulting in incongruence between nuclear and plastid phylogenies [66,67,68]. The cyto-nuclear discordance has been a good first approximation for the detection of reticulate evolution [69]. The incongruence of the topology of the phylogenies revealed in this study indicated that hybridization has occurred in the evolutionary history of *Artemisia*. In fact, morphology also provides evidence for such events. For example, the *Artemisia* species are often widely distributed and almost exclusively wind-pollinated [70]. In the future, single- and low-copy nuclear genes are needed to investigate hybridization in the genus.

The phylogenetic position of *A. stracheyi* Hook. f. & Thomson ex C. B. Clarke is still controversial. It was originally described as a new species in *Artemisia* and recorded to occur in Tibet and adjacent regions [71]. Ghafoor noted that *A. stracheyi* differs remarkably from the genus *Artemisia* in several of its morphological characteristics, including densely scaly corolla and ovary, included stamens, triangular-ovate and obtuse apical anther appendages, as well as quadrangular-pyramidal achenes [72]. They thus proposed a new genus, *Artemisiella* Ghafoor, to accommodate this species and accordingly proposed a new combination, i.e., *Artemisella stracheyi* (C.B. Clarke) Ghafoor. This treatment was not generally accepted by later authors [1,2]. Jiao et al. sampled this species in their phylogenetic study for the first time and found that *Ajania quercifolia* and *Artemisiella stracheyi* formed a clade that is a sister to the genus *Artemisia*. They thus accepted the treatment proposed by Ghafoor [21,50]. In this study, *Artemisiella stracheyi* (=*Artemisia stracheyi*) formed an independent clade within *Artemisia*. But, this clade was weakly supported (BS = 57, PP = 0.89). Morphologically, this species is unique in *Artemisia* by having 2- or 3-pinnatisect leaves with lobules narrowly linear, large (6–10 mm in diam.) involucre, and deciduously pubescent receptacles. The discordance may reflect the complex evolutionary history of *A. stracheyi*. It is highly likely that this species has a hybridization origin. Therefore, in the near future, phylogenetic analyses using plastome and nuclear data with denser sampling and more molecular data, combined with evidence from morphological, cytological, geographical, and ecological studies, are needed to reveal its evolutionary history and determine its phylogenetic position.

As mentioned before, *Artemisia* is such a large, complex, and economically important taxon. It should remain a priority for taxonomical and evolutionary studies, even though these tasks are rather challenging. In this study, we newly sequenced 34 *Artemisia* plastomes, but the taxon sampling is still inadequate, especially the taxa of subg. *Pacifica* and subg. *Tridentatae,* which were mainly distributed in the Hawaiian Islands and North America, respectively. In order to construct a robust phylogeny and to comprehensively reveal the evolutionary history of the genus, future studies should sample more taxa and use more molecular data, especially single-copy nuclear genes.

## Data Availability

The original contributions presented in this study are included in the article/Supplementary Materials. Further inquiries can be directed to the the first author and corresponding author.

## Supplementary Materials

The following supporting information can be downloaded at: https://www.mdpi.com/article/10.3390/genes16060659/s1, Figure S1. Comparison of the borders of large single-copy (LSC), inverted repeat (IR), and small single-copy (SSC) regions among 60 *Artemisia* plastomes. JLB (IRb /LSC), JSB (IRb/SSC), JSA (SSC/IRa), and JLA (IRa/LSC) denote the JSs between each corresponding region in the genome. Figure S2. Maximum likelihood (ML) tree constructed using 79 CDSs of plastomes. Figure S3. Bayesian inference (BI) tree constructed using 79 CDSs of plastomes. Table S1. Information for newly sampled taxa with Genbank accession number for plastome sequences. Table S2. Information for plastomes was downloaded from NCBI. Table S3. The nucleotide variability (Pi) values of protein-coding genes (CDS) and intergenic regions in 72 *Artemisia* plastomes. Table S4. The number of different types of SSR in each plastome.

## Abbreviations

CDS	protein-coding sequences
LSC	large single-copy region
SSC	small single-copy region
IR	inverted repeat region
