# Supplementary figures and images for: Comparative Analysis of Plastomes of Artemisia and Insights into the Infra-Generic Phylogenetic Relationships Within the Genus
Source: Genes (Basel). 2025 May 29;16(6):659. doi: 10.3390/genes16060659 (PMC12192359; doi:10.3390/genes16060659)

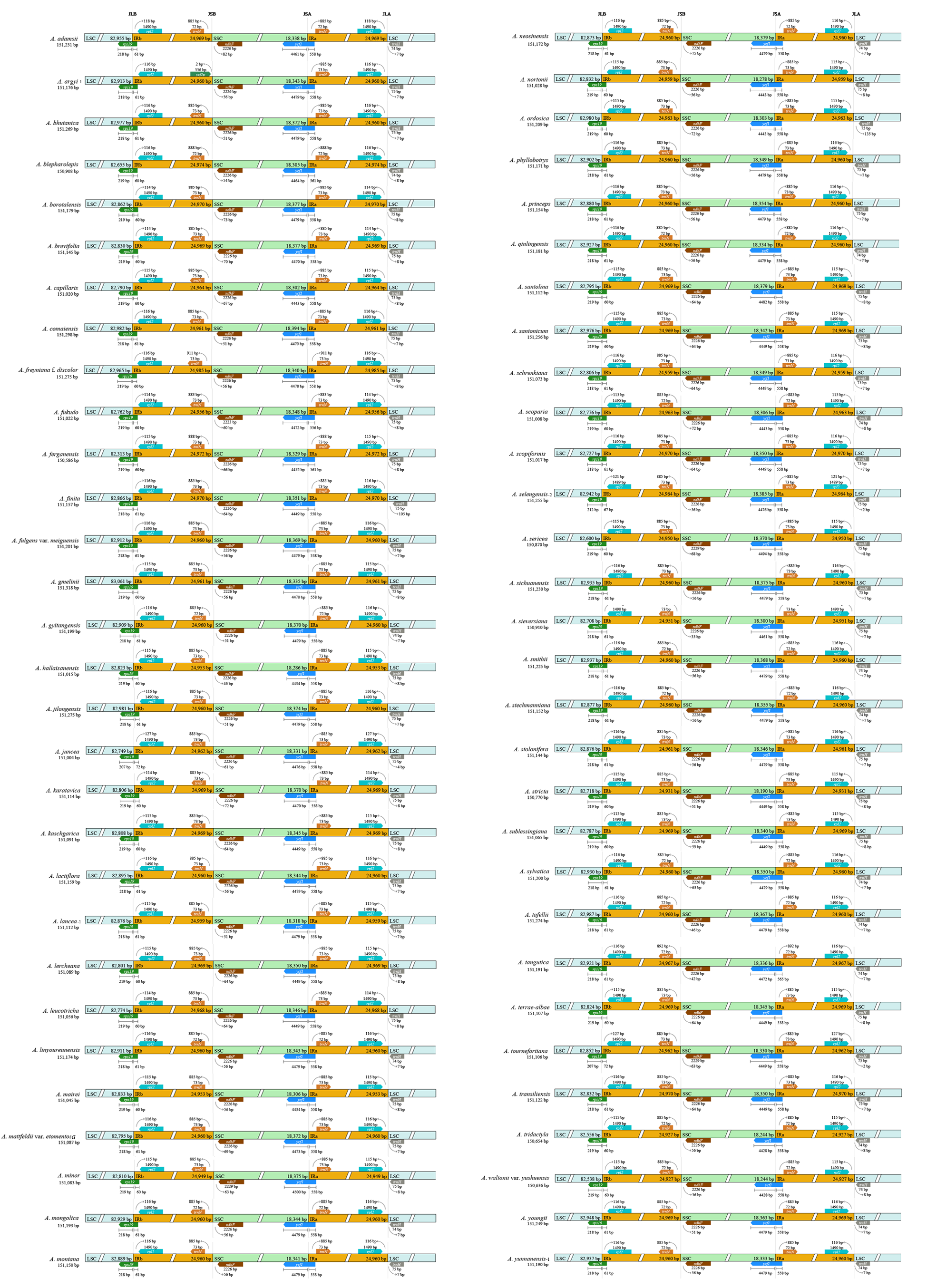

Supplement: Supplementary file 1 [file genes-16-00659-s001.zip › FIGURE S1.tif]

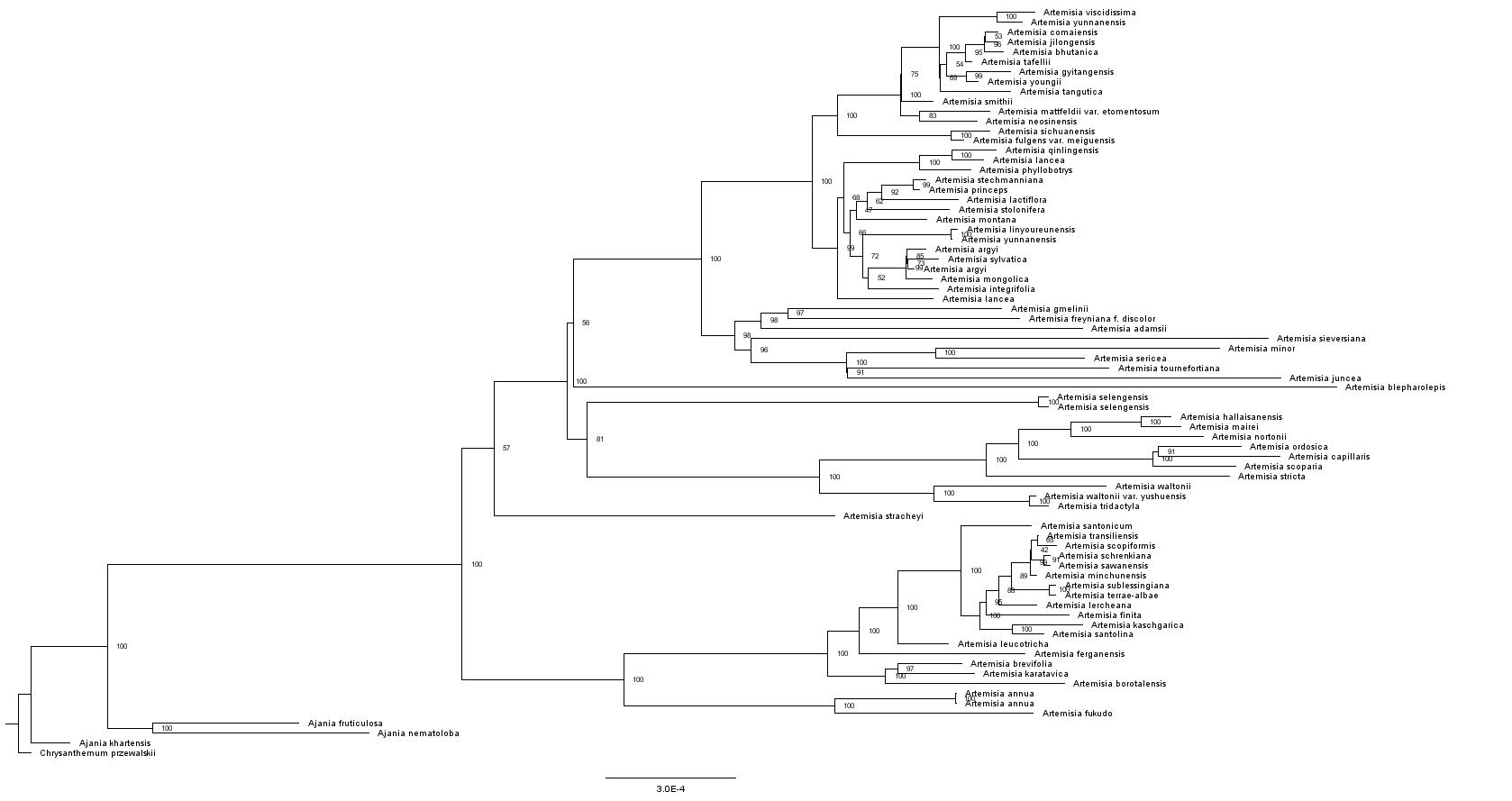

Supplement: Supplementary file 1 [file genes-16-00659-s001.zip › FIGURE S2.jpg]

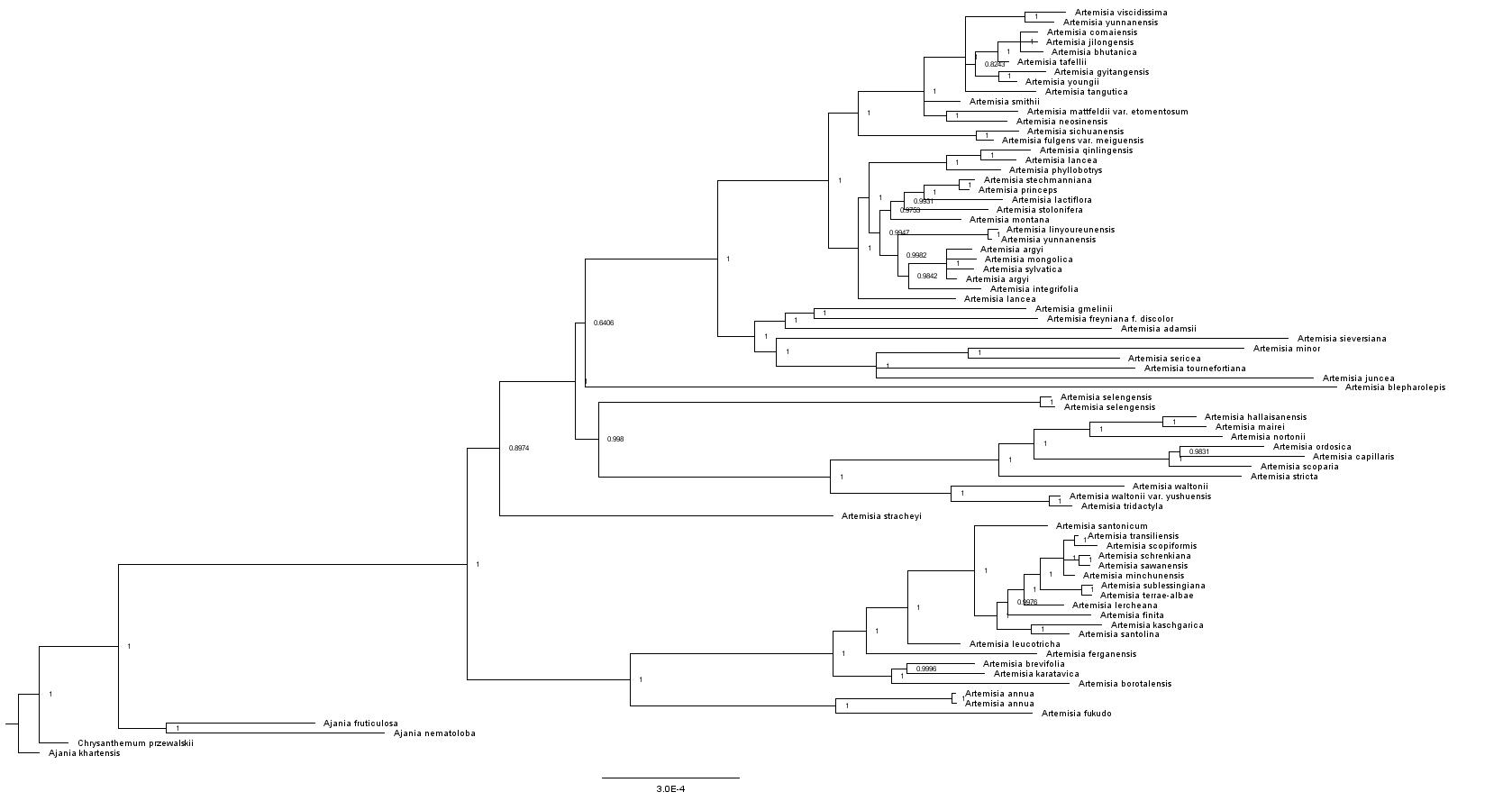

Supplement: Supplementary file 1 [file genes-16-00659-s001.zip › FIGURE S3.jpg]
